# Partnering With African American Churches to Support Families Affected by Dementia Through Research

**DOI:** 10.1093/geroni/igaa057.2776

**Published:** 2020-12-16

**Authors:** Fayron Epps

**Affiliations:** Emory University, Atlanta, Georgia, United States

## Abstract

African Americans (AA) are disproportionately impacted by dementia when compared to the non-Hispanic white population, yet are significantly underrepresented in research. Often times, families in the AA community turn to their church for help when in distress. Recognizing that churches are frequently the cornerstone of AA communities, they are an ideal setting for health promotion, research, and education. However, many AA churches do not have the resources to support their congregants affected by dementia. To build capacity within churches to address brain health promotion and facilitate research access/participation, we partnered with 6 predominantly AA churches in the metropolitan Atlanta area to facilitate research and develop dementia-related programs. While stakeholders were initially reluctant, continual engagement with senior faith leaders helped to facilitate the successful development of a research registry of congregants interested in participating in faith-based and clinical research and establishment of new programs to congregants around brain health and dementia.

